# (*S*)-*N*-(1-Hydroxy­methyl-2-methyl­prop­yl)-2-methoxy­benzamide

**DOI:** 10.1107/S160053680801009X

**Published:** 2008-05-10

**Authors:** Jihong Li, Wenhai Wang, Jingbo Lan, Jingsong You

**Affiliations:** a Key Laboratory of Green Chemistry and Technology of the Ministry of Education, College of Chemistry, Sichuan University, Chengdu 610064, People’s Republic of China

## Abstract

The title compound, C_13_H_19_NO_3_, is an important synthetic inter­mediate. Weak O—H⋯O and N—H⋯O hydrogen bonds enhance the stability of the crystal structure.

## Related literature

For related literature, see: Ma & You (2007 ▶); Rechavi & Lemaire (2002 ▶).
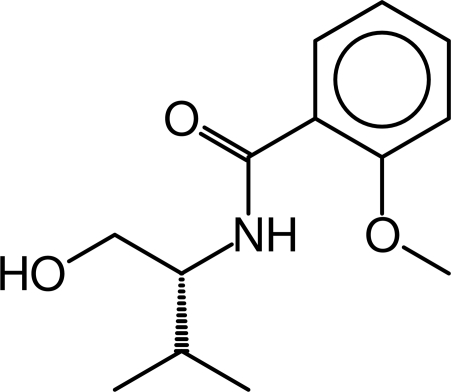


## Experimental

### 

#### Crystal data

C_13_H_19_NO_3_
*M* *_r_* = 237.29
Orthorhombic, 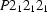

*a* = 9.015 (4) Å
*b* = 10.386 (4) Å
*c* = 14.005 (4) Å
*V* = 1311.3 (9) Å^3^
*Z* = 4
Mo *K*α radiation
μ = 0.09 mm^−1^
*T* = 291 (2) K
0.50 × 0.44 × 0.40 mm

#### Data collection

Enraf–Nonius CAD-4 diffractometer
Absorption correction: none
1457 measured reflections
1397 independent reflections
848 reflections with *I* > 2σ(*I*)
*R*_int_ = 0.010
3 standard reflections every 120 reflections intensity decay: 0.4%

#### Refinement

*R*[*F* ^2^ > 2σ(*F* ^2^)] = 0.045
*wR*(*F* ^2^) = 0.136
*S* = 1.02
1397 reflections
164 parameters
H-atom parameters constrained
Δρ_max_ = 0.21 e Å^−3^
Δρ_min_ = −0.14 e Å^−3^

### 

Data collection: *DIFRAC* (Gabe & White, 1993 ▶); cell refinement: *DIFRAC*; data reduction: *NRCVAX* (Gabe *et al.*, 1989 ▶); program(s) used to solve structure: *SHELXS97* (Sheldrick, 2008 ▶); program(s) used to refine structure: *SHELXL97* (Sheldrick, 2008 ▶); molecular graphics: *ORTEPIII* (Burnett & Johnson, 1996 ▶); software used to prepare material for publication: *SHELXL97*.

## Supplementary Material

Crystal structure: contains datablocks global, I. DOI: 10.1107/S160053680801009X/er2052sup1.cif

Structure factors: contains datablocks I. DOI: 10.1107/S160053680801009X/er2052Isup2.hkl

Additional supplementary materials:  crystallographic information; 3D view; checkCIF report

## Figures and Tables

**Table 1:** Hydrogen-bond geometry (Å, °)

*D*—H⋯*A*	*D*—H	H⋯*A*	*D*⋯*A*	*D*—H⋯*A*
O3—H3⋯O2^i^	0.82	2.00	2.806 (4)	170
N1—H1N1⋯O1	0.86	1.96	2.656 (4)	137

Symmetry code: (i) 
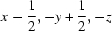
.

**Fig. 1.**
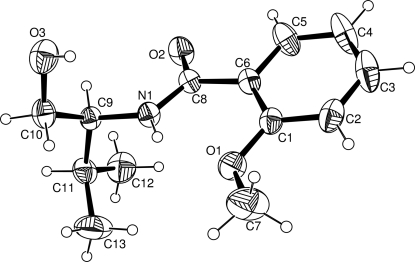
The molecular structure of (I), showing 30% probability displacement ellipsoids and the atomic numbering.

## supplementary crystallographic information

### Comment

Oxazoline ligands have been proved to be a class of chiral ligands, being capable of forming a broad variety of metal complexes that are capable of catalyzing a great number of reactions with excellent enantioselectivity (Rechavi & Lemaire, 2002). It is believed that the oxazoline ring can be modified structurally by replacing the O atom with a substituted N atom, leading to new types of imidazoline ligands (Ma & You, 2007). However, all those ligands can prepared by this compound as an intermediate. Herein, we report the synthesis and structure of the title compound (I).

As shown in Fig. 1, there is a chiral center at C9 derived from *L*-valinol. The C—N bond lengths are 1.318 (4) Å and 1.463 (4) Å, and the C8—N1—C9 angle is 125.3 (3) °. A combination of O—H···O and N—H···O hydrogen bonds interactions provide packing forces in the crystal structure of the title compound.

### Experimental

NaH (8.7 g, 60%, 0.216 mol) was added portionwise to a stirred solution of *L*-valinol (22.1 g, 0.215 mol) in dry THF (120 ml). The mixture was stirred at ambient temperature for 1 h. To this solution was added 2-Methoxy-benzoic acid methyl ester (17.8 g, 0.107 mol) dissolved in THF (50 ml). The mixture was refluxed for 12 h under nitrogen, quenched with H_2_O (10 ml) and concentrated by evaporation of the solvent. The residue was dissolved in CH_2_Cl_2_ (100 ml), washed with H_2_O, brine, and dried over MgSO_4_. And then removal of the solvent *in vacuo* gave a white solid, which was recrystallized from ethyl acetate and petroleum ether to afford the title compound as white crystals (22.8 g, 90%).

### Refinement

H atoms were positioned geometrically and refined in the riding model approximation with O—H = 0.82 Å, N—H = 0.86 Å, and C—H = 0.93, 0.96, 0.97 or 0.98 Å. The *U*_iso_(H) = 1.5 *U*_eq_(C) for the CH_3_ while it was set to 1.2 *U*_eq_(C,*N*,*O*) for all other H atoms. Due to abscence of significant anomalous dispersion effects, the reflection data were merged.

### Crystal data

**Table:**

C_13_H_19_NO_3_	*F*_000_ = 512
*M**_r_* = 237.29	*D*_x_ = 1.202 Mg m^−^^3^
Orthorhombic, *P*2_1_2_1_2_1_	Mo *K*α radiation λ = 0.71073 Å
Hall symbol: P 2ac 2ab	Cell parameters from 25 reflections
*a* = 9.015 (4) Å	θ = 4.5–6.7º
*b* = 10.386 (4) Å	µ = 0.09 mm^−^^1^
*c* = 14.005 (4) Å	*T* = 291 (2) K
*V* = 1311.3 (9) Å^3^	Block, colourless
*Z* = 4	0.50 × 0.44 × 0.40 mm

### Data collection

**Table:**

Enraf–Nonius CAD-4 diffractometer	*R*_int_ = 0.010
Radiation source: fine-focus sealed tube	θ_max_ = 25.5º
Monochromator: graphite	θ_min_ = 2.4º
*T* = 291(2) K	*h* = −3→10
ω/2θ scans	*k* = −3→12
Absorption correction: none	*l* = −5→16
1457 measured reflections	3 standard reflections
1397 independent reflections	every 120 reflections
848 reflections with *I* > 2σ(*I*)	intensity decay: 0.4%

### Refinement

**Table:**

Refinement on *F*^2^	Hydrogen site location: inferred from neighbouring sites
Least-squares matrix: full	H-atom parameters constrained
*R*[*F*^2^ > 2σ(*F*^2^)] = 0.045	*w* = 1/[σ^2^(*F*_o_^2^) + (0.0778*P*)^2^ + 0.0096*P*] where *P* = (*F*_o_^2^ + 2*F*_c_^2^)/3
*wR*(*F*^2^) = 0.136	(Δ/σ)_max_ < 0.001
*S* = 1.02	Δρ_max_ = 0.21 e Å^−^^3^
1397 reflections	Δρ_min_ = −0.14 e Å^−^^3^
164 parameters	Extinction correction: SHELXL97 (Sheldrick, 2008), Fc^*^=kFc[1+0.001xFc^2^λ^3^/sin(2θ)]^-1/4^
Primary atom site location: structure-invariant direct methods	Extinction coefficient: 0.069 (8)
Secondary atom site location: difference Fourier map	

### Special details

**Table:**

Geometry. All e.s.d.'s (except the e.s.d. in the dihedral angle between two l.s. planes) are estimated using the full covariance matrix. The cell e.s.d.'s are taken into account individually in the estimation of e.s.d.'s in distances, angles and torsion angles; correlations between e.s.d.'s in cell parameters are only used when they are defined by crystal symmetry. An approximate (isotropic) treatment of cell e.s.d.'s is used for estimating e.s.d.'s involving l.s. planes.
Refinement. Refinement of *F*^2^ against ALL reflections. The weighted *R*-factor *wR* and goodness of fit *S* are based on *F*^2^, conventional *R*-factors *R* are based on *F*, with *F* set to zero for negative *F*^2^. The threshold expression of *F*^2^ > σ(*F*^2^) is used only for calculating *R*-factors(gt) *etc*. and is not relevant to the choice of reflections for refinement. *R*-factors based on *F*^2^ are statistically about twice as large as those based on *F*, and *R*- factors based on ALL data will be even larger.

### Fractional atomic coordinates and isotropic or equivalent isotropic displacement parameters (Å^2^)

**Table:**

	*x*	*y*	*z*	*U*_iso_*/*U*_eq_	
O1	0.1432 (3)	0.3872 (3)	0.16126 (19)	0.0666 (8)	
O2	0.5192 (3)	0.3286 (3)	−0.00264 (18)	0.0703 (8)	
O3	0.2632 (4)	0.0046 (3)	−0.0111 (2)	0.0810 (10)	
H3	0.1945	0.0562	−0.0140	0.097*	
N1	0.3536 (3)	0.2340 (2)	0.0926 (2)	0.0487 (8)	
H1N1	0.2711	0.2433	0.1227	0.058*	
C1	0.1842 (4)	0.4786 (3)	0.0963 (3)	0.0514 (9)	
C2	0.1074 (5)	0.5935 (4)	0.0851 (3)	0.0709 (12)	
H2	0.0240	0.6099	0.1223	0.085*	
C3	0.1535 (6)	0.6826 (4)	0.0201 (4)	0.0878 (16)	
H3A	0.1009	0.7591	0.0133	0.105*	
C4	0.2760 (6)	0.6610 (4)	−0.0355 (4)	0.0929 (18)	
H4	0.3078	0.7226	−0.0791	0.112*	
C5	0.3519 (5)	0.5457 (4)	−0.0255 (3)	0.0745 (13)	
H5	0.4337	0.5300	−0.0642	0.089*	
C6	0.3096 (4)	0.4533 (3)	0.0402 (3)	0.0489 (9)	
C7	0.0018 (6)	0.3961 (7)	0.2048 (3)	0.109 (2)	
H7A	−0.0038	0.4739	0.2416	0.163*	
H7B	−0.0130	0.3233	0.2460	0.163*	
H7C	−0.0735	0.3970	0.1564	0.163*	
C8	0.4020 (4)	0.3328 (3)	0.0425 (2)	0.0459 (9)	
C9	0.4288 (4)	0.1097 (3)	0.1012 (2)	0.0456 (8)	
H9	0.5050	0.1060	0.0514	0.055*	
C10	0.3202 (5)	0.0025 (3)	0.0824 (3)	0.0609 (10)	
H10A	0.3691	−0.0794	0.0932	0.073*	
H10B	0.2387	0.0091	0.1273	0.073*	
C11	0.5075 (5)	0.0978 (4)	0.1981 (3)	0.0632 (11)	
H11	0.5515	0.0115	0.2002	0.076*	
C12	0.6339 (6)	0.1925 (5)	0.2074 (4)	0.0939 (16)	
H12A	0.5957	0.2787	0.2045	0.141*	
H12B	0.7031	0.1794	0.1562	0.141*	
H12C	0.6832	0.1796	0.2674	0.141*	
C13	0.4068 (6)	0.1084 (6)	0.2833 (3)	0.107 (2)	
H13A	0.4630	0.0946	0.3406	0.160*	
H13B	0.3299	0.0447	0.2788	0.160*	
H13C	0.3631	0.1926	0.2849	0.160*	

### Atomic displacement parameters (Å^2^)

**Table:**

	*U*^11^	*U*^22^	*U*^33^	*U*^12^	*U*^13^	*U*^23^
O1	0.0560 (16)	0.0735 (18)	0.0704 (16)	0.0177 (16)	0.0102 (13)	0.0020 (15)
O2	0.0553 (16)	0.0652 (18)	0.0903 (18)	−0.0022 (15)	0.0249 (16)	0.0137 (16)
O3	0.072 (2)	0.071 (2)	0.099 (2)	0.0007 (16)	−0.0180 (18)	−0.0113 (17)
N1	0.0369 (15)	0.0481 (16)	0.0610 (17)	0.0057 (15)	0.0078 (14)	0.0039 (14)
C1	0.050 (2)	0.046 (2)	0.058 (2)	−0.0001 (18)	−0.0117 (19)	−0.0054 (18)
C2	0.064 (3)	0.060 (3)	0.089 (3)	0.015 (2)	−0.016 (2)	−0.019 (2)
C3	0.070 (3)	0.048 (2)	0.146 (4)	0.002 (2)	−0.043 (3)	0.008 (3)
C4	0.067 (3)	0.059 (3)	0.152 (5)	−0.011 (3)	−0.030 (3)	0.047 (3)
C5	0.053 (2)	0.066 (2)	0.104 (3)	−0.010 (2)	−0.011 (2)	0.031 (3)
C6	0.044 (2)	0.0442 (18)	0.059 (2)	−0.0051 (17)	−0.0149 (17)	0.0013 (17)
C7	0.077 (3)	0.156 (6)	0.094 (3)	0.036 (4)	0.030 (3)	0.020 (4)
C8	0.037 (2)	0.046 (2)	0.054 (2)	−0.0041 (17)	−0.0023 (16)	0.0044 (18)
C9	0.0387 (18)	0.0447 (19)	0.0534 (19)	0.0069 (17)	0.0040 (15)	−0.0004 (17)
C10	0.053 (2)	0.050 (2)	0.080 (3)	0.0044 (19)	0.000 (2)	0.004 (2)
C11	0.062 (3)	0.061 (3)	0.067 (2)	0.016 (2)	−0.009 (2)	0.011 (2)
C12	0.096 (3)	0.093 (3)	0.093 (3)	0.000 (3)	−0.037 (3)	−0.011 (3)
C13	0.120 (4)	0.146 (5)	0.054 (2)	0.031 (5)	0.004 (3)	0.016 (3)

### Geometric parameters (Å, °)

**Table:**

O1—C1	1.365 (4)	C6—C8	1.504 (5)
O1—C7	1.416 (5)	C7—H7A	0.9600
O2—C8	1.232 (4)	C7—H7B	0.9600
O3—C10	1.406 (5)	C7—H7C	0.9600
O3—H3	0.8200	C9—C10	1.506 (5)
N1—C8	1.318 (4)	C9—C11	1.537 (5)
N1—C9	1.463 (4)	C9—H9	0.9800
N1—H1N1	0.8600	C10—H10A	0.9700
C1—C2	1.389 (5)	C10—H10B	0.9700
C1—C6	1.402 (5)	C11—C13	1.503 (6)
C2—C3	1.363 (6)	C11—C12	1.511 (6)
C2—H2	0.9300	C11—H11	0.9800
C3—C4	1.369 (7)	C12—H12A	0.9600
C3—H3A	0.9300	C12—H12B	0.9600
C4—C5	1.386 (6)	C12—H12C	0.9600
C4—H4	0.9300	C13—H13A	0.9600
C5—C6	1.383 (5)	C13—H13B	0.9600
C5—H5	0.9300	C13—H13C	0.9600
			
C1—O1—C7	119.1 (4)	N1—C8—C6	118.4 (3)
C10—O3—H3	109.5	N1—C9—C10	109.7 (3)
C8—N1—C9	125.3 (3)	N1—C9—C11	111.0 (3)
C8—N1—H1N1	117.4	C10—C9—C11	113.3 (3)
C9—N1—H1N1	117.4	N1—C9—H9	107.5
O1—C1—C2	122.5 (4)	C10—C9—H9	107.5
O1—C1—C6	117.5 (3)	C11—C9—H9	107.5
C2—C1—C6	120.0 (4)	O3—C10—C9	112.9 (3)
C3—C2—C1	120.4 (4)	O3—C10—H10A	109.0
C3—C2—H2	119.8	C9—C10—H10A	109.0
C1—C2—H2	119.8	O3—C10—H10B	109.0
C2—C3—C4	121.0 (4)	C9—C10—H10B	109.0
C2—C3—H3A	119.5	H10A—C10—H10B	107.8
C4—C3—H3A	119.5	C13—C11—C12	109.8 (4)
C3—C4—C5	118.8 (4)	C13—C11—C9	114.6 (3)
C3—C4—H4	120.6	C12—C11—C9	111.8 (3)
C5—C4—H4	120.6	C13—C11—H11	106.7
C6—C5—C4	122.0 (5)	C12—C11—H11	106.7
C6—C5—H5	119.0	C9—C11—H11	106.7
C4—C5—H5	119.0	C11—C12—H12A	109.5
C5—C6—C1	117.7 (4)	C11—C12—H12B	109.5
C5—C6—C8	116.0 (3)	H12A—C12—H12B	109.5
C1—C6—C8	126.2 (3)	C11—C12—H12C	109.5
O1—C7—H7A	109.5	H12A—C12—H12C	109.5
O1—C7—H7B	109.5	H12B—C12—H12C	109.5
H7A—C7—H7B	109.5	C11—C13—H13A	109.5
O1—C7—H7C	109.5	C11—C13—H13B	109.5
H7A—C7—H7C	109.5	H13A—C13—H13B	109.5
H7B—C7—H7C	109.5	C11—C13—H13C	109.5
O2—C8—N1	122.0 (3)	H13A—C13—H13C	109.5
O2—C8—C6	119.6 (3)	H13B—C13—H13C	109.5
			
C7—O1—C1—C2	13.4 (5)	C9—N1—C8—C6	179.2 (3)
C7—O1—C1—C6	−167.0 (4)	C5—C6—C8—O2	9.9 (5)
O1—C1—C2—C3	179.3 (3)	C1—C6—C8—O2	−171.7 (3)
C6—C1—C2—C3	−0.3 (6)	C5—C6—C8—N1	−169.6 (3)
C1—C2—C3—C4	−0.0 (6)	C1—C6—C8—N1	8.8 (5)
C2—C3—C4—C5	0.9 (7)	C8—N1—C9—C10	−130.9 (4)
C3—C4—C5—C6	−1.5 (7)	C8—N1—C9—C11	103.2 (4)
C4—C5—C6—C1	1.2 (6)	N1—C9—C10—O3	63.2 (4)
C4—C5—C6—C8	179.7 (4)	C11—C9—C10—O3	−172.2 (3)
O1—C1—C6—C5	−179.8 (3)	N1—C9—C11—C13	59.7 (4)
C2—C1—C6—C5	−0.2 (5)	C10—C9—C11—C13	−64.2 (5)
O1—C1—C6—C8	1.8 (5)	N1—C9—C11—C12	−66.1 (4)
C2—C1—C6—C8	−178.6 (3)	C10—C9—C11—C12	170.0 (3)
C9—N1—C8—O2	−0.3 (5)		

### Hydrogen-bond geometry (Å, °)

**Table:**

*D*—H···*A*	*D*—H	H···*A*	*D*···*A*	*D*—H···*A*
O3—H3···O2^i^	0.82	2.00	2.806 (4)	170
N1—H1N1···O1	0.86	1.96	2.656 (4)	137

Symmetry codes: (i) *x*−1/2, −*y*+1/2, −*z*.
